# The influence of salivary contamination during light curing on degree of conversion and color stability of two composite resins

**DOI:** 10.1016/j.jobcr.2025.12.009

**Published:** 2025-12-24

**Authors:** Marzieh Rohaninasab, Shima Falahat, Golnaz Tayebi, Farzaneh Manouchehri, Farzaneh Sadeghi Mahounak

**Affiliations:** a Laser Research Center of Dentistry, Dentistry Research Institute, Tehran University of Medical Sciences, Tehran, Iran; b School of Dentistry, International Campus, Tehran University of Medical Sciences, Tehran, Iran; c Department of Dental Biomaterials, School of Dentistry, Tehran University of Medical Sciences, Tehran, Iran; d Department of Restorative Dentistry, School of Dentistry, Tehran University of Medical Sciences, Tehran, Iran

**Keywords:** Saliva, Curing lights, Dental, Composite resin, Color stability

## Abstract

**Background:**

Adequate polymerization of composite resin restorations is critical for their mechanical performance and long-term clinical success. Salivary contamination during light curing can interfere with polymerization and may influence color stability.

**Objective:**

This in vitro study examined how artificial saliva contamination at specific intervals during light curing affects the DC (degree of conversion) and short-term color change or ΔE (Delta E) of two composite resins—Gradia Direct (microhybrid) and N-Ceram Bulk Fill (nanohybrid).

**Methods:**

One hundred eighty disk specimens (n = 90 per composite) were allocated into nine experimental groups differing in contamination timing and curing duration. DC was determined by FTIR-ATR (Fourier-Transform Infrared- Attenuated Total Reflectance) spectroscopy immediately after curing and following 24-h incubation at 37 °C. Color change (ΔE) was measured with a spectrophotometer using the CIE (International Commission on Illumination) L*a*b∗ system. Statistical analysis employed one- and two-way ANOVA (Analysis of Variance) with Tukey's post hoc test (α = 0.05).

**Results:**

Early contamination (within the first 5–10 s) produced a significant reduction in DC for both materials (p < 0.001), with N-Ceram showing the greatest loss. Gradia achieved higher DC across most conditions. Post-cure incubation improved DC in all groups, most notably in N-Ceram. All ΔE values remained below the perceptibility threshold of 3.3, with no significant differences among groups (p > 0.05).

**Conclusion:**

Saliva exposure early in light curing markedly reduces polymerization efficiency, although extended curing and post-cure polymerization can partially restore DC. Short-term color stability appears unaffected. Strict field isolation and optimized curing protocols are essential to maximize clinical performance.

## Graphical abstract

**Figure:**
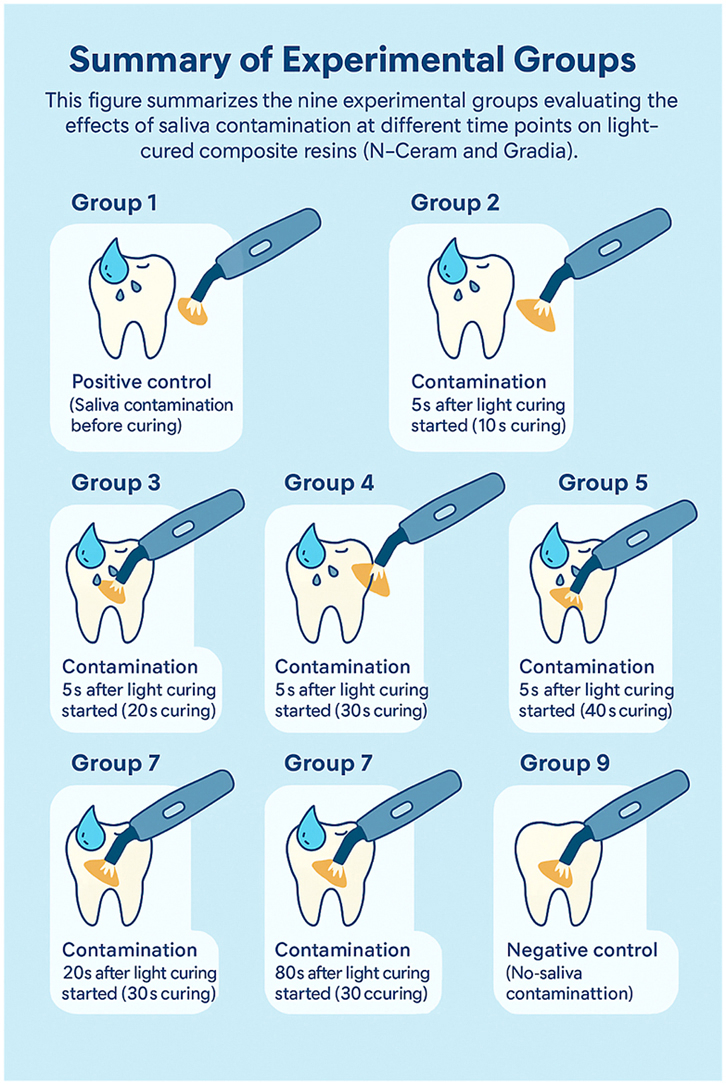

## 1 Introduction

Composite resins remain the cornerstone of contemporary restorative dentistry due to their excellent esthetic qualities, reliable adhesion, and compatibility with minimally invasive techniques. Continuous developments in filler technology, resin matrices, photoinitiators, and light-curing devices have significantly enhanced the mechanical and optical properties of these materials. Nevertheless, limitations such as polymerization shrinkage and incomplete curing still compromise their long-term clinical performance.^1^

The degree of conversion (DC)—the proportion of carbon–carbon double bonds converted into single bonds during polymerization—serves as a fundamental indicator of composite quality. Higher DC values correlate with improved mechanical strength, reduced water sorption, and favorable biocompatibility, whereas lower DC values contribute to residual monomer release, accelerated degradation, and reduced service longevity.^1^

Numerous factors influence DC, including the composite formulation (e.g., monomer chemistry, filler characteristics, viscosity), layer thickness, light intensity, and exposure duration.^2^ Among these, environmental contamination during clinical procedures is often underestimated. Salivary contamination, in particular, is a frequent occurrence when isolation is challenging, as in posterior teeth, deep cavities, or pediatric cases.^3^

Saliva is a complex biological fluid rich in water, proteins, glycoproteins, enzymes, and electrolytes. Owing to its composition, it may interfere with polymerization by diluting or scattering the curing light and suppressing free-radical propagation.^4^ Contamination can occur before, during, or immediately after light curing, potentially altering surface properties and curing efficiency.^5^

Earlier and recent research have explored the influence of contamination timing on DC and mechanical parameters.^5^^,^^6^ Systematic evaluations indicate that both saliva and blood exposure can reduce adhesive performance, with decontamination protocols showing only partial recovery.^6^ Furthermore, saliva substitutes have been reported to affect color stability more noticeably than water immersion,^7^ and incidental contamination from saliva or gloves has been associated with altered surface morphology and microhardness, particularly in nanohybrid composites.^8^ Polymerization kinetics analysis also highlights the role of extended curing time in improving DC and minimizing residual monomer content, especially in bulk-fill materials.^9^

Despite these advances, comprehensive in-vitro evidence integrating the variables of contamination timing, material type, and curing duration remains limited. To address this gap, the present study investigates the effect of artificial saliva contamination introduced at defined intervals during the light-curing process on the DC and short-term color stability (ΔE) of two commercially available composite resins—a microhybrid (Gradia Direct) and a bulk-fill nanohybrid (N-Ceram). Measurements were performed immediately after curing and following a 24-h incubation period.

## 2 Methods

### 2.1 Study design and ethics approval

This in vitro experimental study investigated the effect of artificial saliva contamination at different stages of light curing on the degree of conversion (DC) and short-term color stability (ΔE) of two light-cured composite resins. The study protocol was approved by the Ethics Committee of Tehran University of Medical Sciences (Approval Code: IR. TUMS.DENTISTRY.REC1403.015). All procedures were performed in accordance with manufacturer guidelines for material handling.

### 2.2 Sample size calculation

Sample size estimation was based on data from *Aidaros* et al.^10^ using G∗Power software (version 3.1, Heinrich Heine University, Düsseldorf, Germany). For ΔE evaluation, with a power of 80 % (β = 0.2), α = 0.05, a standard deviation of 2.2, and an effect size of 0.42, the minimum required sample size was 10 specimens per group. For DC, the anticipated effect size was 1.0 with a standard deviation of 2.1, indicating that as few as three specimens would be adequate; however, for consistency across all tests, each group comprised 10 specimens per composite resin.

### 2.3 Materials

Two commercially available, light-cured composite resins were tested:

• **Gradia Direct™** (GC Corporation, Tokyo, Japan) – microhybrid composite, shade A2.
• **N-Ceram Bulk Fill™** (Ivoclar Vivadent, Liechtenstein) – nanohybrid bulk-fill composite, shade A2.

Material compositions are summarized in Supplementary Table 1. All products were stored at room temperature and used before their expiration dates.

### 2.4 Specimen fabrication

A total of 180 disc-shaped specimens (n = 90 per composite) were fabricated using a custom-made silicone mold (4 mm diameter × 2 mm thickness). Each mold was filled incrementally with the assigned composite, covered with a transparent polyester strip, and pressed flat using a glass slide to eliminate voids and standardize thickness.

### 2.5 Grouping and experimental conditions

Specimens for each composite were randomly assigned to nine experimental groups (n = 10 per group) according to contamination timing and total curing time (Table 1):

**Table 1:** Description of nine experimental groups.

Group	Contamination Timing	Total Curing Time	Description
G1 – PC/SBC	Before curing	40s (N-Ceram), 20s (Gradia)	Positive control with pre-cure contamination
G2 – S5C10	5s after light started	10s total	Early contamination with short curing
G3 – S5C20	5s after light started	20s total	Early contamination with medium curing
G4 – S5C30	5s after light started	30s total	Early contamination with extended curing
G5 – S5C40	5s after light started	40s total	Early contamination with full curing
G6 – S10C20	10s after light started	20s total	Mid-curing contamination
G7 – S20C30	20s after light started	30s total	Late-stage contamination
G8 – S30C40	30s after light started	40s total	Very late contamination
G9 – NC	No contamination	40s (N-Ceram), 20s (Gradia)	Negative control (ideal condition)

G1 – PC/SBC: Positive Control/Saliva Contamination Before Curing, G2 – S5C10: Saliva contamination 5s after start light curing, total Curing time 10s, G3 – S5C20: Saliva contamination 5s after start light curing, total Curing time 20s, G4 – S5C30: Saliva contamination 5s after start light curing, total Curing time 30s, G5 – S5C40: Saliva contamination 5s after start light curing, total Curing time 40s, G6 – S10C20: Saliva contamination 10s after start light curing, total Curing time 20s, G7 – S20C30: Saliva contamination 20s after start light curing, total Curing time 30s, G8 – S30C40: Saliva contamination 30s after start light curing, total Curing time 40s, G9 – NC: Negative Control.

### 2.6 Artificial saliva preparation

Artificial saliva was prepared according to the Fusayama–Meyer formula, consisting of:

• NaCl (0.4 g),
• KCl (1.21 g),
• NaH_2_PO_4_·2H_2_O (0.78 g),
• Na_2_S·9H_2_O (0.005 g), and
• Urea (1.0 g),

per 1000 mL of deionized water, with pH adjusted to 6.7 at 25 °C.

### 2.7 Contamination protocol

At the assigned contamination time, 100 μL of artificial saliva was applied to the composite surface using a sterile insulin syringe. For the pre-cure contamination group (G1), saliva was placed on the uncured composite immediately before light activation. For other groups, curing was briefly paused at the designated time to allow saliva application, after which curing resumed until the planned total duration was reached.

### 2.8 Light-curing procedure

All polymerization was performed with a calibrated polywave LED curing unit (Bluephase G2, Ivoclar Vivadent, Liechtenstein) delivering 1200 mW/cm^2^. The light tip was positioned 1 mm above the specimen surface using a custom jig to maintain consistent distance and angle. Gradia was cured according to manufacturer recommendations (20 s) except where extended curing times were specified. N-Ceram was cured for up to 40 s as per group assignment.

### 2.9 Degree of conversion (DC) measurement

DC was measured using Fourier-transform infrared spectroscopy with attenuated total reflectance (FTIR-ATR; Avatar 370, Thermo Nicolet, USA). A small portion of uncured composite was analyzed to obtain a reference spectrum. Each cured specimen was then placed directly on the ATR crystal for spectral acquisition.

The DC (%) was calculated using the following formula^3^:

DC (%) = [1 – (R_cured / R_uncured)] × 100

where **R** is the ratio of absorbance peaks at 1638 cm^−1^ (aliphatic C=C) and 1608 cm^−1^ (aromatic C–C).^3^ Measurements were recorded immediately after curing and after 24 h storage in distilled water at 37 °C.

### 2.10 Color stability (ΔE) measurement

Color was measured with a calibrated spectrophotometer (VITA EasyShade® Advance) under standardized lighting and a neutral gray background. CIE L*a*b∗ values were recorded before curing (**L_0_, a_0_, b_0_**) and after 24-h incubation (**L_1_, a_1_, b_1_**). ΔE was calculated as:

ΔE = √[(ΔL)^2^ + (Δa)^2^ + (Δb)^2^]

where ΔL = L_1_ – L_0_, Δa = a_1_ – a_0_, and Δb = b_1_ – b_0_. A ΔE value of 3.3 was considered the perceptibility threshold for clinical relevance.

### 2.11 Statistical analysis

Data were analyzed using SPSS Statistics v27 (IBM Corp., Armonk, NY, USA). Normality was assessed using the Shapiro–Wilk test. One-way ANOVA with Tukey's post hoc test was applied for intergroup comparisons within each material. Two-way ANOVA assessed the interaction between contamination timing and curing duration. Statistical significance was set at **p < 0.05**.

## 3 Results

### 3.1 Specimen integrity and dataset overview

All 180 specimens (90 Gradia, 90 N-Ceram) were successfully fabricated and analyzed. No samples were lost or damaged during curing, storage, or measurement. Results are presented separately for degree of conversion (DC) and color stability (ΔE).

### 3.2 Degree of conversion (DC)

**Fig. 1:**
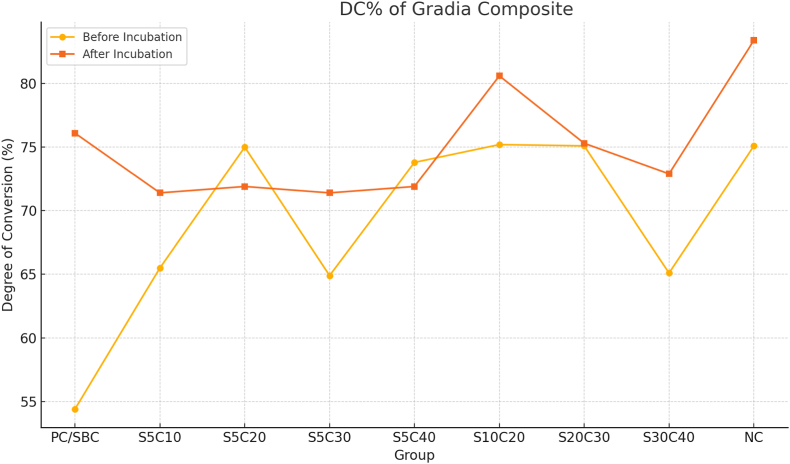
DC% of gradia Composite.

**Fig. 2:**
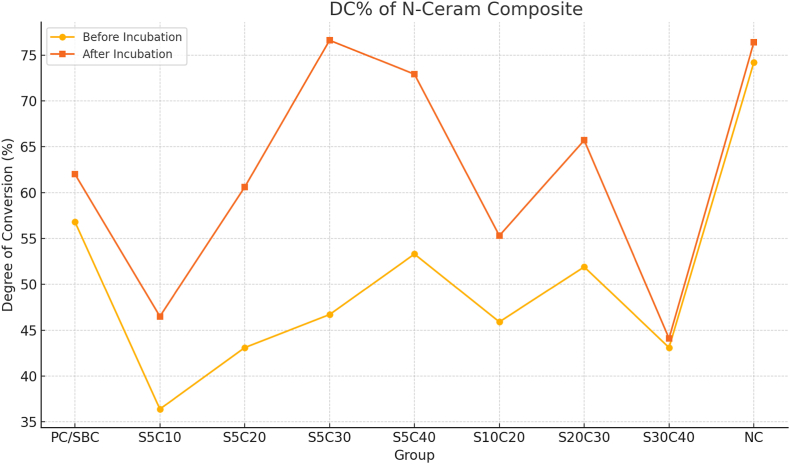
DC% of N-ceram Composite.

The mean of DC values of Gradia and N-Ceram specimens before and after incubation are shown in Fig. 1, Fig. 2. In general, Gradia exhibited higher DC values than N-Ceram across most experimental conditions.

### 3.3 Overall trends

Across most experimental conditions, Gradia achieved higher DC values than N-Ceram. Early salivary contamination, particularly within the first 5–10 s of curing, produced the greatest reduction in DC for both materials.

### 3.4 Effect of contamination timing and curing duration

One-way ANOVA demonstrated significant differences in DC among contaminated groups and the uncontaminated control for both composites (p < 0.001).

**Gradia:** Post-incubation DC in group S10C20 (saliva at 10 s, total cure 20 s) was statistically comparable to the negative control (p = 0.12).

**N-Ceram:** Post-incubation DC in group S5C30 (saliva at 5 s, total cure 30 s) did not differ significantly from control (p = 0.07).

Two-way ANOVA revealed a significant interaction between contamination timing and curing duration (p < 0.01), indicating that these factors act synergistically in influencing polymerization efficiency.

### 3.5 Effect of post-cure incubation

After 24 h of storage in distilled water at 37 °C, DC increased in all groups, consistent with ongoing polymerization of residual monomers. This effect was more pronounced in N-Ceram than in Gradia.

However, in groups with severe early contamination (e.g., N-Ceram S5C10), post-cure incubation did not fully restore DC, which remained substantially below control levels.

### 3.6 Inter-material comparison

Across nearly all conditions, Gradia outperformed N-Ceram. The largest disparity occurred in group S5C10, where Gradia reached 71.4 % DC after incubation compared to only 46.5 % for N-Ceram (p < 0.01). Even in late contamination scenarios such as group S30C40, Gradia maintained a higher DC (72.9 %) than N-Ceram (44.1 %), with the difference remaining statistically significant (p < 0.01).

### 3.7 Color stability (ΔE)

Mean ΔE values ranged from **1.12 to 1.97** for Gradia and **1.20 to 2.11** for N-Ceram.

• One-way ANOVA found no statistically significant differences in ΔE between groups for either material (p > 0.05).
• All ΔE values remained below the perceptibility threshold of 3.3, indicating that contamination timing and curing duration did not cause clinically noticeable discoloration over the short term.

Summary of key findings:

1. Early contamination significantly reduced DC in both composites, with N-Ceram more adversely affected.
2. Gradia consistently achieved higher DC under comparable conditions.
3. Post-cure incubation enhanced DC across all groups, with bulk-fill N-Ceram showing greater relative recovery.
4. ΔE values remained clinically acceptable in all cases, with no significant group differences.

## 4 Discussion

This study examined how salivary contamination at different stages of light curing influences the degree of conversion (DC) and short-term color stability (ΔE) of two composite resins: the microhybrid Gradia Direct and the bulk-fill nanohybrid N-Ceram. The results demonstrated that early contamination—particularly within the first 5–10 s of curing—substantially reduced DC in both materials. Gradia exhibited greater resistance to contamination effects, while post-cure incubation significantly improved DC in both composites, especially N-Ceram. In contrast, short-term color stability was unaffected, with all ΔE values remaining below clinically perceptible thresholds.

### 4.1 Impact of contamination timing on polymerization

Our findings confirm that the earliest phases of light curing are particularly vulnerable to contamination. Groups contaminated within 5–10 s (e.g., S5C10, S10C20) exhibited the largest declines in DC. This agrees with previous studies indicating that uninterrupted light exposure during the initial polymerization phase is critical for optimal free radical formation and network crosslinking.^11^, ^12^, ^13^ Saliva contamination at this stage may lead to the deposition of a proteinaceous layer, which can scatter curing light and inhibit free radical propagation.^14^

These results align with *Lee* et al. (2024),^15^ who observed over a 20 % reduction in DC in nanohybrid composites exposed to saliva during the first 10 s of curing. Similarly, *Ryu* et al. (2025),^16^ in a meta-analysis, identified this early window as the most critical for maintaining polymer integrity.

### 4.2 Material-dependent differences

Gradia consistently achieved higher DC than N-Ceram across contamination scenarios. This is likely attributable to its microhybrid formulation, which features a balanced filler/resin ratio and lower viscosity, allowing better light penetration and more efficient polymerization. Prior research supports this observation, showing that microhybrid composites tend to perform better under compromised curing conditions.^17^^,^^18^

Interestingly, although N-Ceram demonstrated lower initial DC under contamination, it exhibited substantial improvement after 24-h incubation, consistent with the extended polymerization kinetics reported for bulk-fill composites.^19^ Bulk-fill materials often contain alternative photoinitiator systems and higher translucency, enabling post-cure radical activity and delayed conversion gains.^20^^,^^21^

### 4.3 Post-cure incubation effects

The improvement in DC after 24 h was evident for both materials, but more pronounced in N-Ceram. This is consistent with studies showing that unreacted monomers in bulk-fill composites can continue to polymerize for extended periods when stored at elevated temperatures.^20^^,^^21^

Nevertheless, post-cure recovery was incomplete in groups subjected to severe early contamination. For example, N-Ceram in group S5C10 remained far below control levels even after incubation, suggesting that certain early-stage disruptions may permanently limit polymerization potential. This underscores the clinical importance of preventing contamination during the initial curing phase.

### 4.4 Short-term color stability

In this study, all ΔE values remained below the clinical threshold of 3.3, and no significant differences were found between groups or materials. This indicates that short-term discoloration is unlikely to occur solely from salivary contamination during curing. *Wang* et al. (2023)^22^ similarly found that different polymerization profiles did not significantly affect immediate color stability.

However, our results do not rule out potential long-term esthetic changes. *Benetti* et al. (2024)^23^ demonstrated that composites with lower DC values are more susceptible to staining and discoloration over time, especially under exposure to common beverages such as coffee or tea. Thus, while short-term color change was minimal in our study, long-term follow-up under aging conditions may reveal clinically relevant differences.

### 4.5 Clinical implications

The results of this study have several practical takeaways:

1. **Avoid early contamination:** The first 10 s of light curing are critical; any interruption during this period can have lasting effects on polymerization.
2. **Select materials strategically:** Microhybrid composites such as Gradia may offer better resilience when ideal isolation is difficult to achieve.
3. **Mitigate damage if contamination occurs:** Extending curing time and allowing a post-cure period can partially restore DC, particularly in bulk-fill materials, but complete recovery is not guaranteed.
4. **Consider long-term risks:** While short-term color stability may remain unaffected, reduced DC could predispose restorations to future discoloration and mechanical degradation.

## 5 Conclusion

This study demonstrated that salivary contamination during the early stages of light curing significantly compromises the degree of conversion of composite resins. The microhybrid Gradia Direct consistently showed greater tolerance to contamination compared with the bulk-fill nanohybrid N-Ceram. A 24-h post-cure incubation enhanced DC in all groups, particularly in N-Ceram, although early contamination effects were not always fully reversible.

Short-term color stability, as measured by ΔE, was not significantly influenced by contamination timing or composite type, with all values remaining below the clinical perceptibility threshold.

Future investigations should address long-term effects of contamination on color stability, monomer release, and mechanical performance under simulated oral aging.

## Declaration of patient/guardian consent

I hereby certify that informed consent was obtained from all patients (or their legal guardians, in case of minors) whose extracted teeth were used in this study entitled *"[*The Influence of Salivary Contamination During Light Curing on Degree of Conversion and Color Stability of Two Composite Resins: An In Vitro Study*]"*.

Patients/guardians were informed that the extracted teeth would be used solely for research purposes, without any personal identifiers, and that participation was voluntary. Anonymity and confidentiality were strictly maintained throughout the study.

## Ethical approvement

The study protocol was approved by the Ethics Committee of Tehran University of Medical Sciences (Approval Code: IR. TUMS.DENTISTRY.REC1403.015).

## Declaration of generative AI and AI-assisted technologies in the writing process

During the preparation of this work the author(s) used ChatGPT (OpenAI, San Francisco, CA, USA) solely to assist with language refinement and grammar improvement. After using this tool/service, the authors reviewed and edited the content as needed and takes full responsibility for the content of the publication.

## Funding

The authors declare that this study received no funding from any source.

## Declaration of competing interest

The authors declare that they have no known competing financial interests or personal relationships that could have appeared to influence the work reported in this paper.
