# Supplementary material for: The influence of salivary contamination during light curing on degree of conversion and color stability of two composite resins
Source: J Oral Biol Craniofac Res. 2025 Dec 24;16(1):254–9. doi: 10.1016/j.jobcr.2025.12.009 (PMC12800347; doi:10.1016/j.jobcr.2025.12.009)
Supplement: Multimedia component 1 [file mmc1.docx]

| Material | Manufacturer | Composition |
| --- | --- | --- |
| N-Ceram Bulk Fill™ | Ivoclar vivadent AG, Germany | Nanohybrid flowable composite 40 nanosized filler: Barium Aluminum borosilicate, Bis-GMA, UDMA, Bis-EMA, TMPTMA (75 weight %, 37 volume %) |
| Gradia Direct™ | GC, Japan | Crystalline Silica,Silicone dioxide,lithium/barium-aluminium glass,borosilicate glass containing zinc/strontium/lithium,zirconia-silica,or zirconium oxide |
| Artificial saliva | Synthesized in the Dental Biomaterials Laboratory, Iran | Prepared by mixing of 0.4 g sodium chloride (NaCl), 1.21 g potassium chloride (KCl), 0.78 g sodium dihydrogen dehydrate (NaH2PO4.2H2O), 0.005 g hydrated sodium sulfide (Na2S.9H2O), and 1 g urea CO(NH2)2 in 1000 ml deionized water. The pH of this mixture was modified with 10 N sodium hydroxide until it reached 6.7 on a pH meter |
| Alcohol | Central Hamoon Teb, Iran | 70% Ethyl alcohol |
